# Impact of the COVID-19 pandemic on the real-world diagnostic infrastructure for tuberculosis—An ESGMYC collaborative study

**DOI:** 10.1371/journal.pone.0291404

**Published:** 2024-04-16

**Authors:** Laura Paulowski, Roxana Filip, Mateja Jankovic Makek, Lorenzo Guglielmetti, Delia Goletti, Jakko van Ingen, Katharina Kranzer, Florian P. Maurer, on behalf of ESGMYC

**Affiliations:** 1 National and WHO Supranational Reference Laboratory for Mycobacteria, Research Center Borstel, Borstel, Germany; 2 Tuberculosis and Molecular Biology Laboratory, Suceava Emergency County Hospital, Suceava, Romania; 3 Faculty of Medicine and Biological Sciences, Stefan Cel Mare Suceava University, Suceava, Romania; 4 Department for Respiratory Diseases Jordanovac, University Hospital Center Zagreb, Zagreb, Croatia; 5 School of Medicine, University of Zagreb, Zagreb, Croatia; 6 Sorbonne Université, INSERM, U1135, Centre d’Immunologie et des Maladies Infectieuses, Cimi-Paris, Paris, France; 7 APHP, Groupe Hospitalier Universitaire Sorbonne Université, Hôpital Pitié-Salpêtrière, Centre National de Référence des Mycobactéries et de la Résistance des Mycobactéries aux Antituberculeux, Paris, France; 8 Department of Epidemiology and Preclinical Research, National Institute for Infectious Diseases L. Spallanzani-IRCCS, Rome, Italy; 9 Department of Medical Microbiology, Radboud University Medical Center, Nijmegen, The Netherlands; 10 Clinical Research Department, London School of Hygiene and Tropical Medicine, London, United Kingdom; 11 Biomedical Research and Training Institute, Harare, Zimbabwe; 12 Division of Infectious Diseases and Tropical Medicine, University Hospital, LMU Munich, Munich, Germany; 13 University Medical Center Hamburg-Eppendorf, Hamburg, Germany; 14 German Centre for Infection Research (DZIF), Partner Site Hamburg-Lübeck-Borstel-Riems, Hamburg, Germany; Shandong Public Health Clinical Center: Shandong Provincial Chest Hospital, CHINA

## Abstract

We determined the impact of the COVID-19 pandemic on mycobacterial diagnostic services. 40 laboratories from 22 countries completed an online questionnaire covering the redeployment of the laboratory infrastructure and/or staff for SARS-CoV-2 testing, staff shortages and supply chain disruptions. 28 laboratories reported monthly numbers of samples processed for mycobacterial investigations and monthly numbers of *M*. *tuberculosis* complex (MTBC) PCRs performed between October 1st 2018 and October 31st 2020. More than half (23/40) of the participating TB laboratories reported having performed COVID-19 diagnostics in the early phase of the pandemic, in part with negative impact on the mycobacterial service activities. All participating laboratories reported shortages of consumables and laboratory equipment due to supply chain issues. Average monthly sample numbers decreased by 24% between January 2020 and October 2020 compared to pre-pandemic averages. At the end of the study period, most participating laboratories had not returned to pre-pandemic average MTBC PCR throughput.

## Introduction

Tuberculosis (TB) services globally have been disproportionately affected by the coronavirus disease 2019 (COVID-19) pandemic [1]. Before COVID-19, TB was the leading infectious killer of humans, affecting 10 million people in 2019 and causing 1.4 million deaths. However, since the start of the COVID-19 pandemic TB case notifications have substantially declined [2]. COVID-19 directly affected health care delivery due to shortage or redeployment of staff and indirectly because of control measures which led to disrupted supply chains, limited access to healthcare and economic decline. Moreover, since the onset of the COVID-19 pandemic, both TB mortality and estimated incidence have increased for the first time in more than a decade [2,3].

Several studies have investigated the impact of COVID-19 on TB services using TB case notifications as a source [4–7]. However, few studies have examined data from TB laboratory networks [8,9]. While these studies reported disruption of TB diagnostics in the early phase of the COVID-19 pandemic, they are limited by their geographic scope (European Economic Area member states and the World Health Organization European region, respectively) and the type of enrolled laboratories (national reference laboratories only). None of these studies used primary data on the number of samples tested and the number of tests conducted comparing pre-COVID-19 with COVID-19 time periods.

This study aimed to investigate the impact of the COVID-19 pandemic on mycobacterial diagnostic laboratories collaborating within the European Society of Clinical Microbiology and Infectious Diseases (ESCMID) Study Group on Mycobacterial Infections (ESGMYC) and beyond. We aimed in particular to study changes in sample numbers and nucleic acid amplification tests (NAAT) for rapid detection of *Mycobacterium tuberculosis* complex (MTBC) to obtain a quantitative measure of the impact of COVID-19 on mycobacterial diagnostics at an international scale.

## Materials and methods

### Data collection

A two-part online survey was designed using SurveyMonkey (available at https://www.surveymonkey.com/r/COVIDxTB). The first part included eight general questions related to the COVID-19 pandemic covering i) whether the laboratory expanded its scope to SARS-CoV-2 testing, ii) to what extend the service was affected by supply chain disruptions and iii) whether the service was fully operational between October 2018 and October 2020 (see detailed questions in Table 1). In the second part, laboratories were asked to provide monthly numbers of samples received during the study period and monthly numbers of MTBC NAAT performed at the laboratory. The survey was distributed to 154 ESGMYC members and personal contacts of the authors on January 12^th^ 2021. Recipients were invited to further distribute the survey among their networks. A reminder was sent in February 2021. Data collection was closed on September 30^th^ 2021. The authors had no access to information that could identify individual participants during or after data collection except in their own diagnostic services.

**Table 1:** Characterization of the participating laboratories and general impact of the COVID-19 pandemic on 40 tuberculosis diagnostic services. MTBC, *Mycobacterium tuberculosis* complex; SARS-CoV-2, severe acute respiratory syndrome coronavirus type 2; TB, tuberculosis.

Question	N (%)
Question 1: Does your laboratory process primary specimens?	
Yes	35 (87.5)
No	2 (5.0)
No response	3 (7.5)
Question 2: Does your laboratory process MTBC cultures?	
Yes	37 (92.5)
No	0 (0)
No response	3 (7.5)
Question 3: Has your laboratory been involved in SARS-CoV-2 testing?	
Yes	23 (57.5)
No	15 (37.5)
No response	2 (5)
Question 4: Has technical staff of your TB service been involved in SARS-CoV-2 testing?	
Yes, with negative impact on the TB service	5 (21.7^1^)
Yes, without negative impact on the TB service	15 (65.2)
No	3 (13.0)
Question 5: Has academic staff of the TB service been involved in SARS-CoV-2 testing?	
Yes, with negative impact on the TB service	5 (21.7^1^)
Yes, without negative impact on the TB service	12 (52.2)
No	6 (26.1)
Question 6: Has equipment usually used for mycobacteriology testing also been used for SARS-CoV-2 testing?	
Yes, with negative impact on the TB service	5 (21.7^1^)
Yes, without negative impact on the TB service	11 (47.8)
No	7 (30.4)
Question 7: Did you receive any additional resources for SARS-CoV-2 testing?	
Yes, additional funding	15 (65.2^1^)
Yes, additional staff	17 (73.9)
Yes, additional instruments	18 (78.3)
Yes, additional other infrastructure	9 (39.1)
No	1 (4.3)
Question 8: Has any of your staff working in the mycobacteriology service been infected with SARS-CoV-2?	
Yes, <10%	8 (20)
Yes, 10–25%	9 (22.5)
Yes, 25–50%	3 (7.5)
Yes, >50%	0 (0)
No	17 (42.5)
No response	3 (7.5)

^1^ Percentages in relation to those laboratories that confirmed having been involved in SARS-CoV-2 testing in question 3 (n = 23).

### Data analysis

All submitted data were exported from the online survey tool and analyzed using GraphPad Prism software (Version 9.4.1, GraphPad Software, San Diego, CA, USA). Duplicate submissions from the same laboratory were removed. Monthly sample and NAAT numbers processed after the onset of the COVID-19 pandemic, which was defined as January 2020, were compared to the pre-pandemic averages calculated for each laboratory based on data submitted for the period October 2018 until December 2019 and expressed as percentages relative to this average [10]. Color codes used for the heat map representations in Figs 3 and 4 were set as follows: value or range from 0–10% (corresponding to >90% decline compared to the pre-pandemic average), red; 10.01–25% (75–90% decline), orange; 25.01–50% (50–75% decline), yellow; 50.01–75% (25–50% decline), green; 75.01–90% (10–25% decline), blue; 90.01–100% (<10% decline), violet; 100.01–180% (corresponding to a testing increase), light gray.

### Ethics

This study was approved by the Ethics Commission at the University of Lubeck, Germany (reference number 2023–823).

## Results

**Fig 1:**
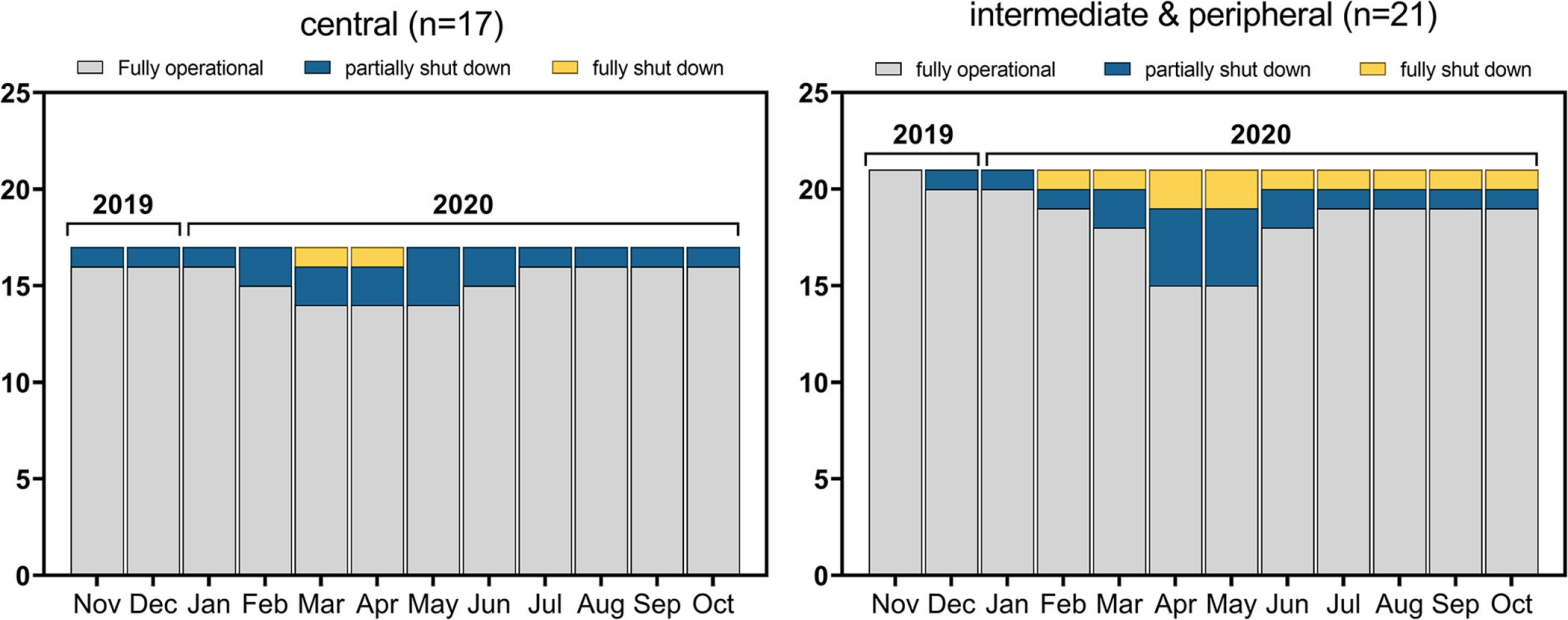
Operational readiness of TB diagnostic services during the first wave of the COVID-19 pandemic. Laboratories were categorized into central, intermediate or peripheral service providers as per WHO definitions [10]. Peripheral laboratories are not shown due to low sample size (n = 2). One laboratory from Zagreb/Croatia reported damages as a result of a regional earthquake during a partial COVID-19 lockdown as the reason for their full shutdown.

**Fig 2:**
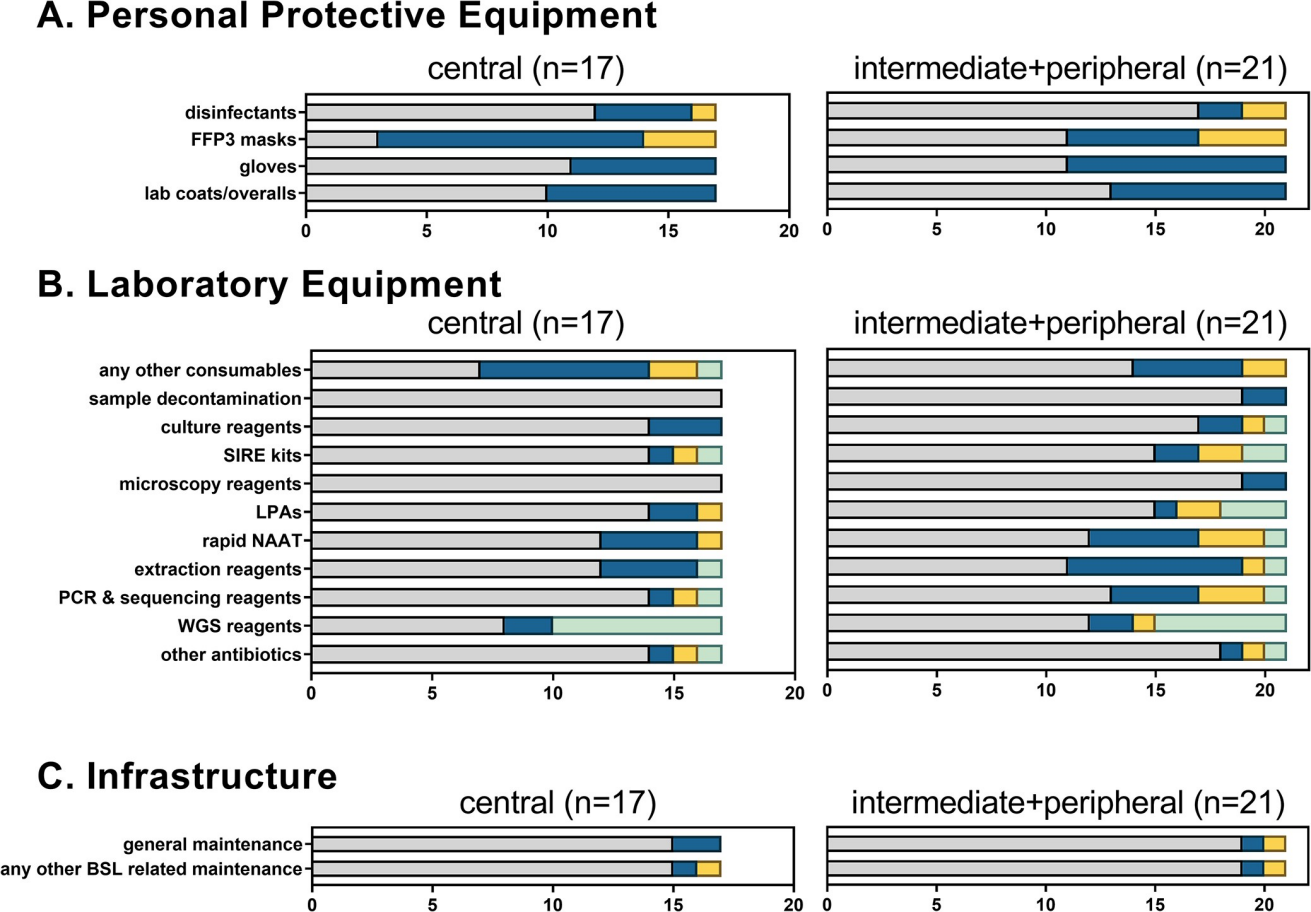
Reagents shortages, supply chain interruptions and impairments in laboratory maintenance at TB diagnostic services during the first wave of the COVID-19 pandemic. BSL, biosafety level 3 laboratory; LPAs, line probe assays; NAAT, nucleic acid amplification tests; PPE, personal protective equipment; SIRE kit, BACTEC™ MGIT™ 960 (Becton Dickinson, Franklin Lakes, NJ, USA) SIRE kit for phenotypic first line susceptibility testing of *Mycobacterium tuberculosis* isolates; WGS, whole genome sequencing.

**Fig 3:**
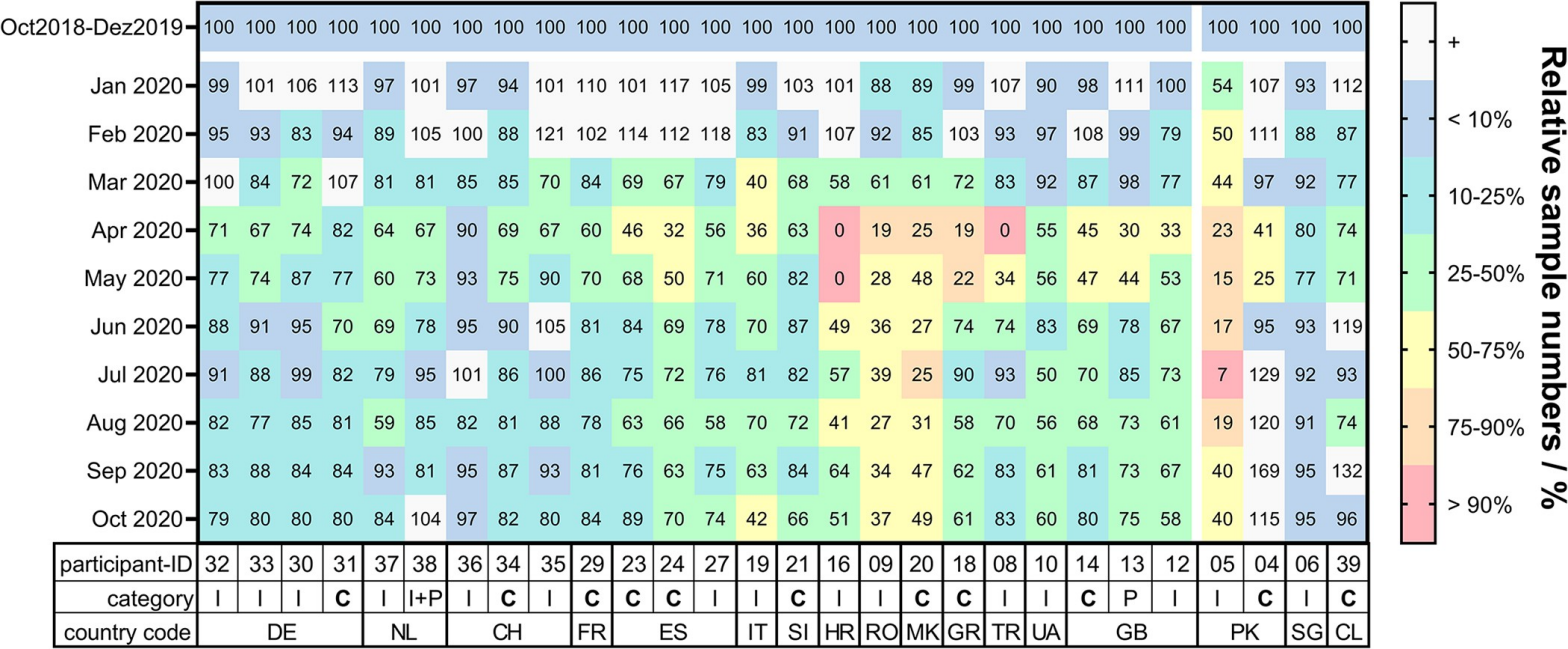
Changes in overall sample numbers received at 28 TB diagnostic services since the onset of the COVID-19 pandemic as compared to the pre-pandemic average. Data reported for 2020 are shown in percent relative to the monthly average of samples received between October 2018 and December 2019. I, intermediate level laboratory; C, central (reference) level laboratory; P, peripheral laboratory. DE, Germany; NL, the Netherlands; CH, Switzerland; FR, France; ES, Spain; IT, Italy; SI, Slovenia; HR, Croatia; RO, Romania; MK, North Macedonia; GR, Greece; TR, Turkey; UA, Ukraine; GB, Great Britain; PK, Pakistan; SG, Singapore; CL, Chile.

**Fig 4:**
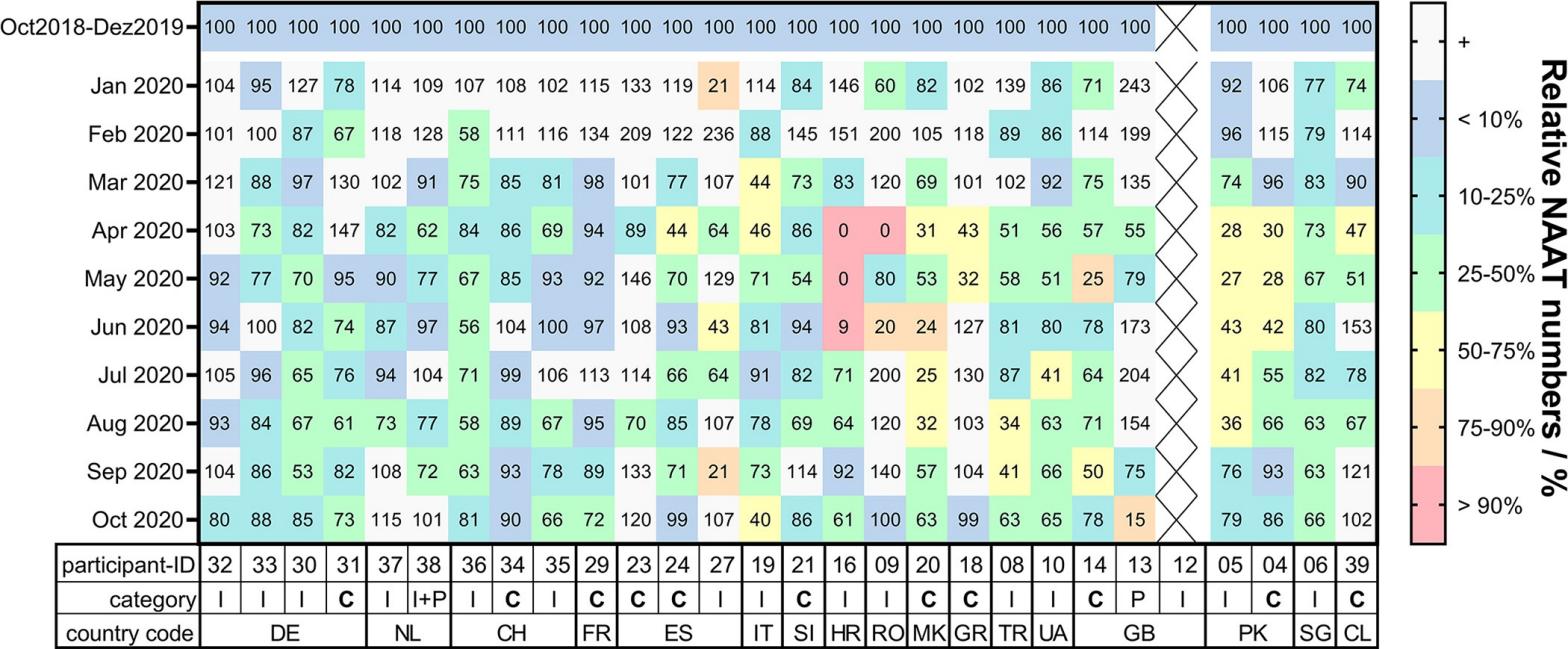
Changes in MTBC nucleic acid amplification tests performed at 28 TB diagnostic services since the onset of the COVID-19 pandemic as compared to the pre-pandemic level. Data reported for 2020 are shown as percent relative to the monthly average of samples received between October 2018 and December 2019. I, intermediate level laboratory; C, central (reference) level laboratory; P, peripheral laboratory. DE, Germany; NL, the Netherlands; CH, Switzerland; FR, France; ES, Spain; IT, Italy; SI, Slovenia; HR, Croatia; RO, Romania; MK, North Macedonia; GR, Greece; TR, Turkey; UA, Ukraine; GB, Great Britain; PK, Pakistan; SG, Singapore; CL, Chile.

In total, 40 laboratories responded to the survey (S1 Table). The laboratories were located in Africa (Mozambique, n = 1), Asia (India, n = 2; Pakistan, n = 2; Saudi Arabia, n = 1; and Singapore, n = 1), Europe (Belgium, n = 1; Croatia, n = 3; France, n = 1; Germany, n = 4; Greece, n = 1; Italy, n = 1; The Netherlands, n = 2; North Macedonia, n = 1; Romania, n = 1; Slovenia, n = 1; Spain, n = 6; Sweden, n = 1; Switzerland, n = 3; Turkey, n = 1; United Kingdom, n = 3; and Ukraine, n = 1), and South America (Chile, n = 2) and included 17 central (reference level), 21 intermediate (regional and district levels), and 2 peripheral (subdistrict and community levels) laboratories [11]. A total of 38 laboratories answered the qualitative part of the survey, while 28 provided quantitative data on monthly sample numbers and MTBC NAAT (Table 1, Figs 1–4).

Most laboratories reported regularly receiving both primary samples for culture and/or NAAT (n = 35/40) and mycobacterial cultures for identification and drug susceptibility testing (n = 37/40, no response, n = 3; Table 1). More than half of the participating laboratories (n = 23/40) indicated that COVID-19 testing was performed in 2020. Five laboratories (India, n = 2; Spain, n = 3), reported that COVID-19 testing negatively affected the mycobacterial diagnostic service (Table 1). Most laboratories involved in COVID-19 testing received additional resources, including staff (n = 17/23), funding (n = 15/23), equipment (n = 18/23), and/or infrastructure (n = 9/23). The proportion of laboratory staff infected with SARS-CoV-2 varied across laboratories: <10%, 10–25% and 25–50% in 8, 9 and 3 laboratories, respectively (Table 1). Most laboratories (31/40) reported that they were able to fully continue with standard operations despite the constraints imposed by the pandemic for the most part between November 2019 and October 2020 (Fig 1). One central laboratory in Spain, and one intermediate level laboratory in each Croatia and India reported that they had to shut down the service for a minimum of one month during 2020. Notably, the full shutdown reported by the laboratory in Croatia was related to a severe earthquake on March 22nd 2020 when the country was in a COVID-19 lockdown [12]. In addition, five laboratories from Spain (n = 2), Turkey, India and Ukraine reported partial shutdowns of at least one month following the onset of the COVID-19 pandemic (Fig 1).

Shortages of consumables and laboratory equipment were reported by all laboratories, though with variable impact (Fig 2). Shortage of disinfectants and FFP3 masks were a common theme in laboratories in Europe, while supplies of other personal protective equipment such as gloves and laboratory coats were not affected. Shortages of consumables typically used for mycobacterial and TB diagnostics were reported more often by intermediate and peripheral laboratories than by central level laboratories (Fig 2). Critical shortages affected all reagent categories including nucleic acid extraction reagents, PCR and DNA sequencing chemicals, culture reagents needed for phenotypic drug susceptibility testing as well as rapid, kit- or cartridge-based nucleic-acid amplification tests.

On average, monthly sample numbers decreased by 9%, 39%, 24%, and 25% in the first, second, third and fourth quarter of 2020, respectively, as compared to the pre-pandemic average (Figs 3 and S1). Declines in sample numbers during the second quarter of 2020 were particularly pronounced in laboratories from Croatia (intermediate, 84%), Romania (intermediate, 72%), North Macedonia (central, 67%), Greece (central, 62%), Turkey (intermediate, 64%), and Pakistan (intermediate, 82%). For one laboratory in Pakistan (ID-05), this corresponds to sample declines from 416 (pre-pandemic monthly average) to a minimum of 29 samples in July 2020 (Fig 3). Interestingly, another laboratory in Pakistan (central, ID-04) reported a short-term 59–75% decrease in April and May 2020, respectively, followed by a phase of above average sample numbers until October 2020 (Fig 3). The laboratory in Kharkiv / Ukraine (ID-10, Fig 3) reported a reduction in samples received of almost 50% in July 2020 with sample numbers remaining low until the end of the study period (60% of the pre-pandemic average in October 2020). The participating laboratories from Singapore and Chile experienced sample declines on par with Western Europe with 16% (Singapore, ID-06) and 12% (Chile, ID-39) fewer samples received in the second quarter of 2020. Sample numbers were back to pre-COVID-19 levels in October 2020 in those laboratories. Overall, 13/28 laboratories reported average sample numbers of less than 80% of the pre-pandemic average at the end of the study period (Fig 3).

The number of MTBC NAAT performed per month fluctuated during the study period and between laboratories from the same countries. In the participating laboratories from Western Europe, we observed declines between 15–47% as well as increases of up to 147% compared to the pre-pandemic averages (Fig 4). Among participants from South and Eastern Europe, the reported numbers fluctuated even stronger, for example in a central laboratory in Greece where MTBC NAAT decreased by 32% compared to the pre-pandemic level in May 2020 followed by an immediate rebound to 127% in June 2020 (Fig 4). Like the monthly number of samples received at the participating laboratory from Ukraine, the number of MTBC NAAT that were performed at this site dropped by almost 60% in July 2020 and remained at relatively low levels until the end of the study period (63–66% of the pre-pandemic average, Fig 4). While not as pronounced as with the overall sample numbers, we also observed a steep decline in MTBC NAAT performed at the two participating sites in Pakistan with minima of 27% and 28%, respectively, in May 2020 as compared to the pre-pandemic level. The site in Singapore reported monthly NAAT throughputs of 63% to 83% as compared to the pre-pandemic average. NAAT numbers at the site in Chile fluctuated considerably between 71% and 132%. Similar to the overall sample numbers received, 12/28 sites in total reported an MTBC NAAT throughput of less than 80% at the end of the study period as compared to the pre-pandemic average.

## Discussion

This is the first study to investigate the impact of the COVID-19 pandemic on mycobacterial diagnostic services based on a multinational analysis of changes in the numbers of samples received in central, intermediate, and peripheral laboratories globally. Our main findings are that the number of samples submitted for mycobacterial investigations declined steeply, particularly during the first months of the COVID-19 pandemic. This effect was less consistent for MTBC NAAT. However, NAAT throughput also remained below the pre-pandemic average at the end of the study period. In addition, we found that most laboratories experienced shortages in personal protective equipment or essential reagents at some point after the onset of the pandemic. These findings illustrate the challenges specialized laboratory services faced in the early phase of the COVID-19 pandemic.

The relative importance of factors contributing to the reduction in sample volumes was likely different in lower and higher resource settings. In low- and middle-income countries, lock-downs resulted in restricted access to public transport and rapidly depleting personal resources due to loss of income [13]. This, in turn, led to a situation in which patients sought help only after a delay or not at all. Notably, disruption of public transport not only affected patients but also healthcare workers and sample transport. Moreover, stock-outs were not only affecting laboratory consumables but also sampling consumables such as sputum pots and personal protective equipment used in clinics. Lastly, overlapping symptoms may have resulted in underdiagnosis of TB as patients with respiratory symptoms may have been assumed to suffer from COVID-19 without further testing for TB [13]. In higher income countries, a reduced influx of migrants from high incidence settings due to closed borders may have played an additional role. Lastly, the relative proportion of samples obtained from patients suspected of being infected with non-tuberculous mycobacteria (NTM) was likely larger at mycobacteriology services in low TB prevalence settings. Routine follow-ups of these patients may have been frequently postponed either because the risk of contracting COVID-19 was thought to be too high, or because the available workforce was heavily engaged in the COVID-19 response. In such settings, less frequent sampling of patients with NTM disease for culture-based follow-ups may also explain why the overall number of samples submitted for mycobacterial diagnostics (including culture) was consistently lower after the onset of the pandemic as compared to pre-pandemic throughputs while a more heterogeneous pattern was observed for MTBC NAAT.

We did not generate direct evidence that the decreased sample throughputs observed in this study resulted in a reduction of TB diagnoses. However, globally, the number of TB notifications decreased in 2020 and TB-related mortality increased in 2021 [3]. As diagnostic services play a crucial role as entry points into care and treatment, we believe that the observed sample declines indeed resulted in fewer diagnoses and, hence, delayed or inadequate treatment.

Our findings point towards several elements of the TB diagnostic cascade that were particularly vulnerable during the COVID-19 pandemic and that may be at risk again during similar events. Firstly, supply chains are prone to disruptions due to closed borders and lockdowns of production sites and transportation hubs such as harbors and airports. Laboratories can mitigate this risk, at least to some extent, by keeping enough critical reagents and consumables in stock and by not exclusively relying on single suppliers. However, the diagnostic mycobacteriology market is dominated by few manufacturers making it difficult to implement backup strategies. Other commercial entities could seize this opportunity to provide a wider choice of alternatives for critical products. A second important learning from the COVID-19 pandemic is that staff shortages can quickly become a serious threat to the continuity of diagnostic services, especially as many laboratories are already having difficulties to recruit skilled personnel in the absence of a pandemic. Laboratories should hence invest in continued education of their workforce as a means to retain their staff and to foster flexible skillsets that allow for a quick reallocation to the services most needed. Larger emergency staff pools comprising not only members of the microbiology service but also those of related disciplines such as clinical chemistry or pathology could also support flexible reallocation in case of a public health crisis. Moreover, laboratory directors should identify opportunities to simplify their diagnostic processes to reduce the need for extensive training, for example by replacing labor-intensive laboratory-developed tests with equivalent assays that are easier to operate. Lastly, automatization of standard processes, such as nucleic acid extraction, can be critical to respond to peak demands, in particular when the laboratory workforce is affected by a public health crisis itself.

This study has some strengths and weaknesses. Firstly, while we aimed to reach wide geographical coverage, most of the participating sites are located in Europe and the limited number of participating laboratories per country, in particular from high TB burden settings, did not allow us to report data that are representative on a country or regional level. However, while some participants did not provide sample numbers, we believe that the quantitative data reported herein, which cover a period of over two years, consistently support the conclusion that COVID-19 severely disturbed the TB laboratory diagnostic sector at an international scale. In this regard, the contributions by non-reference level laboratories present a strength of this study. While we were successful in recruiting a significant number of intermediate level laboratories, the overall number of peripheral laboratories was low. This does not come as a surprise as ESGMYC members mostly represent diagnostic centers. More research will be needed to assess the impact of disruptive events such as pandemics or armed conflicts on decentralized laboratories and peripheral testing sites.

In summary, we performed a two-tiered survey and established that there was a steep decline in the number of samples submitted for mycobacterial diagnostics among an international panel of participating laboratories, particularly during the first months of the COVID-19 pandemic. In addition, we found an overall shortfall in the number of MTBC NAAT that were performed in 2020 likely resulting in delayed TB diagnoses. Most of the participating laboratories experienced shortages in personal protective equipment or essential reagents at some point during the early phase of the COVID-19 pandemic. In consequence, future efforts should place a particular focus on building resilient supply chains and robust pre-analytical sampling and transportation networks to allow diagnostic services to operate at full capacity when it is needed most [14].

## Supporting information

S1 Fig Quarterly changes in overall sample numbers received at 26 TB diagnostic services since the onset of the COVID-19 pandemic as compared to the pre-pandemic average. Data reported for 2020 are shown as percent relative to the monthly average of samples received between October 2018 and December 2019. I, intermediate level laboratory; C, central (reference) level laboratory; P, peripheral laboratory. DE, Germany; NL, the Netherlands; CH, Switzerland; FR, France; ES, Spain; IT, Italy; SI, Slovenia; HR, Croatia; RO, Romania; MK, North Macedonia; GR, Greece; TR, Turkey; UA, Ukraine; GB, Great Britain; PK, Pakistan; SG, Singapore; CL, Chile.
(TIF)

S1 Table Overview of the laboratories participating in the survey. Laboratories are subdivided according to location, laboratory category and the maximum and minimum total monthly numbers of samples received at the laboratory. In addition, it is noted whether participants took part in both parts of the survey or only answered questions 1–8 (Table 1) without providing sample numbers. C, central (reference) level laboratory; I, intermediate level laboratory; N/a, not available; P, peripheral laboratory.
(DOCX)
