# Supplementary figures and images for: Impact of the COVID-19 pandemic on the real-world diagnostic infrastructure for tuberculosis—An ESGMYC collaborative study
Source: PLoS One. 2024 Apr 16;19(4):e0291404. doi: 10.1371/journal.pone.0291404 (PMC11020973; doi:10.1371/journal.pone.0291404)

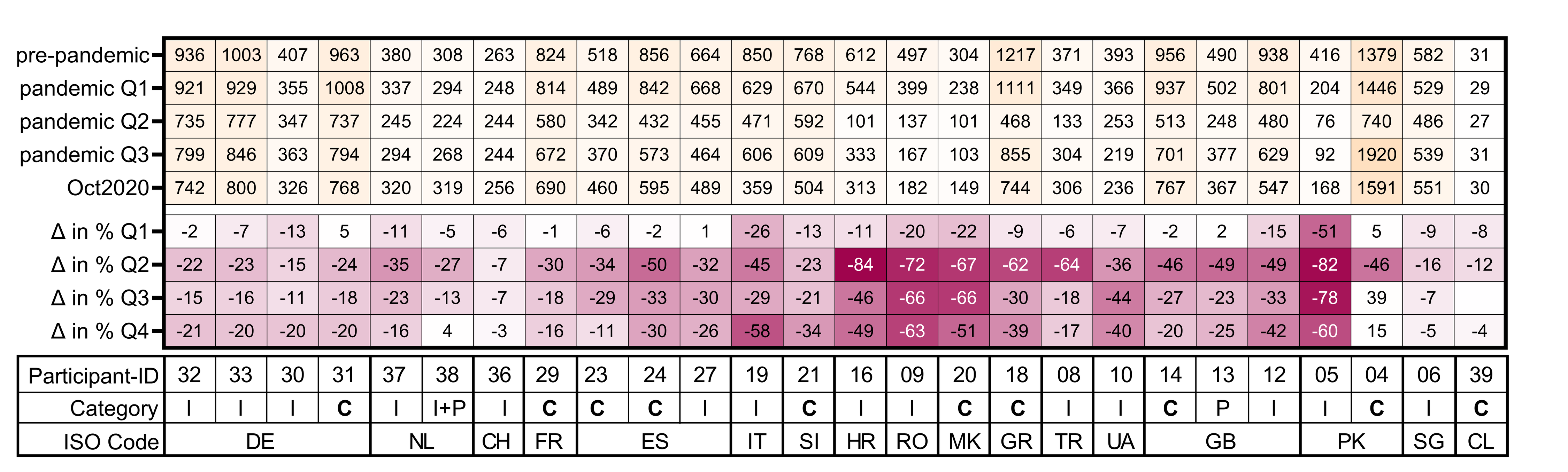

Supplement: S1 Fig — Data reported for 2020 are shown as percent relative to the monthly average of samples received between October 2018 and December 2019. I, intermediate level laboratory; C, central (reference) level laboratory; P, peripheral laboratory. DE, Germany; NL, the Netherlands; CH, Switzerland; FR, France; ES, Spain; IT, Italy; SI, Slovenia; HR, Croatia; RO, Romania; MK, North Macedonia; GR, Greece; TR, Turkey; UA, Ukraine; GB, Great Britain; PK, Pakistan; SG, Singapore; CL, Chile. (TIF) [file pone.0291404.s001.tif]
