# Supplementary material for: Impact of the COVID-19 pandemic on the real-world diagnostic infrastructure for tuberculosis—An ESGMYC collaborative study
Source: PLoS One. 2024 Apr 16;19(4):e0291404. doi: 10.1371/journal.pone.0291404 (PMC11020973; doi:10.1371/journal.pone.0291404)
Supplement: S1 Table — Laboratories are subdivided according to location, laboratory category and the maximum and minimum total monthly numbers of samples received at the laboratory. In addition, it is noted whether participants took part in both parts of the survey or only answered questions 1–8 (Table 1) without providing sample numbers. C, central (reference) level laboratory; I, intermediate level laboratory; N/a, not available; P, peripheral laboratory. (DOCX) [file pone.0291404.s002.docx]

**S1 Table. Overview of the laboratories participating in the survey.** Laboratories are subdivided according to location, laboratory category and the maximum and minimum total monthly numbers of samples received at the laboratory. In addition, it is noted whether participants took part in both parts of the survey or only answered questions 1-8 (Table 1) without providing sample numbers. C, central (reference) level laboratory; I, intermediate level laboratory; N/a, not available; P, peripheral laboratory.

| **ID** | **Participant** | **Region** | **Country** | **ISO Code** | **City** | **Cate-gory** | **Total samples Min** | **Total samples Max** | **Questions answered** | **Sample numbers provided** |
| --- | --- | --- | --- | --- | --- | --- | --- | --- | --- | --- |
| **1** | Laboratorio Nacional de Referencia da Tuberculose | Southern Africa | Mozambique | MZ | Maputo | C | n/a | n/a | yes | no |
| **2** | AIIMS Rishikesh TB lab | South-central Asia | India | IN | Rishikesh | I | n/a^a^ | n/a | yes | no |
| **3** | TB Laboratory, AIIMS, Bhopal | South-central Asia | India | IN | Bhopal | I | n/a | n/a | yes | no |
| **4** | National Tuberculosis Control Program Pakistan | South-central Asia | Pakistan | PK | Islamabad | C | 346 | 2335 | yes | yes |
| **5** | Clinical Laboratories, The Indus Hospital | South-central Asia | Pakistan | PK | Karachi | I | 29 | 754 | no | yes |
| **6** | National University Hospital | South-east Asia | Singapore | SG | Singapore | I | 450 | 641 | yes | yes |
| **7** | Mycobacteriology Laboratory at King Faisal Specialist Hospital & Research Center | Middle East | Saudi Arabia | SA | Riyadh | I | n/a | n/a | yes | no |
| **8** | Marmara University Pendik Hospital, Microbiology Laboratory | Middle East | Turkey | TR | Istanbul | I | 0 | 450 | yes | yes |
| **9** | Mycobacterium and Molecular Biology Laboratory, Suceava Emergency County Hospital | Eastern Europe | Romania | RO | Suceava | I | 93 | 639 | yes | yes |
| **10** | Regional Clinical Tuberculosis Dispensarу #7 | Eastern Europe | Ukraine | UA | Kharkiv | I | 198 | 458 | yes | yes |
| **11** | Linköping | Northern Europe | Sweden | SE | Linköping | I | n/a | n/a | yes | no |
| **12** | Barts Health NHS Trust | Northern Europe | UK, England | GB | London | I | 311 | 1198 | yes | yes |
| **13** | London North West University Healthcare NHS Trust | Northern Europe | UK, England | GB | London | P | 146 | 559 | yes | yes |
| **14** | Scottish Mycobacteria Reference Laboratory | Northern Europe | UK, Scotland | GB | Edinburgh | C | 430 | 1120 | yes | yes |
| **15** | National Reference Laboratory for Tuberculosis | Southern Europe | Croatia | HR | Zagreb | C | n/a | n/a | yes | no |
| **16** | Department of Clinical and Molecular Microbiology, University Hospital Centre Zagreb | Southern Europe | Croatia | HR | Zagreb | I | 0^a^ | 719 | yes | yes |
| **17** | University Hospital of Split | Southern Europe | Croatia | HR | Split | I | n/a | n/a | yes | no |
| **18** | Microbiology Laboratory and National Reference Centre for Mycobacteria, Sotiria Chest Diseases Hospital | Southern Europe | Greece | GR | Athens | C | 237 | 1415 | yes | yes |
| **19** | Laboratory of Microbiology and biorepository, L. Spallanzani National Institute for Infectious Diseases (INMI) | Southern Europe | Italy | IT | Rome | I | 302 | 948 | yes | yes |
| **20** | National Reference Laboratory for Tuberculosis, Institute for Lung Diseases and Tuberculosis | Southern Europe | North Macedonia | MK | Skopje | C | 75 | 368 | yes | yes |
| **21** | Laboratory for Mycobacteria Golnik | Southern Europe | Slovenia | SI | Golnik | C | 485 | 885 | yes | yes |
| **22** | Fundacion Jimenez Diaz | Southern Europe | Spain | ES | Madrid | C | n/a | n/a | yes | no |
| **23** | Microbiology Department, Hospital Universitary of Bellvitge | Southern Europe | Spain | ES | Barcelona | C | 238 | 644 | yes | yes |
| **24** | Mycobacteriology Unit. Hospital Vall d'Hebron de Barcelona | Southern Europe | Spain | ES | Barcelona | C | 277 | 998 | yes | yes |
| **25** | Sección de Micobacterias. Servicio de Microbiología. Hospital Universitario Virgen del Rocío | Southern Europe | Spain | ES | Seville | C | n/a^a^ | n/a | yes | no |
| **26** | Hospital Clinic of Barcelona | Southern Europe | Spain | ES | Barcelona | I | n/a | n/a | yes | no |
| **27** | Servicio de Microbiologia, Hospital Universitario Miguel Servet | Southern Europe | Spain | ES | Zaragoza | I | 374 | 799 | yes | yes |
| **28** | Sciensano | Western Europe | Belgium | BE | Brussels | C | n/a | n/a | yes | no |
| **29** | Institut des Agents Infectieux, LBMMS, Hospices Civils de Lyon | Western Europe | France | FR | Lyon | C | 497 | 944 | yes | yes |
| **30** | Institute for Medical Microbiology and Hospital Epidemiology, Hannover Medical School | Western Europe | Germany | DE | Hannover | C | 295 | 465 | yes | yes |
| **31** | National and WHO Supranational Reference Center for Mycobacteria | Western Europe | Germany | DE | Borstel | C | 679 | 1089 | yes | yes |
| **32** | MVZ am Helios Klinikum Emil von Behring | Western Europe | Germany | DE | Berlin | I | 661 | 1062 | no | yes |
| **33** | Labor Berlin, Charité und Vivantes GmbH | Western Europe | Germany | DE | Berlin | I | 673 | 1243 | yes | yes |
| **34** | National Laboratory for Mycobacteriology, Institute of Medical Microbiology | Western Europe | Switzerland | CH | Zurich | C | n/​a | n/a | yes | yes |
| **35** | Institute of Microbiology, Lausanne University Hospital | Western Europe | Switzerland | CH | Lausanne | I | n/a | n/a | yes | yes |
| **36** | Mycobacteriology Laboratory, Division for Clinical Bacteriology and Mycology, University Hospital Basel | Western Europe | Switzerland | CH | Basel | I | 215 | 310 | yes | yes |
| **37** | Radboudumc | Western Europe | The Netherlands | NL | Nijmegen | I | 229 | 438 | yes | yes |
| **38** | Medical Microbiology and Infectious Diseases | Western Europe | The Netherlands | NL | Rotterdam | I/P | 207 | 359 | yes | yes |
| **39** | Laboratorio Clínico Clínica Alemana de Santiago | South America | Chile | CL | Santiago | C | 21 | 44 | yes | yes |
| **40** | National and WHO Supranational Reference Laboratory for Mycobacteria | South America | Chile | CL | Santiago | C | n/a | n/a | yes | no |

^a^ These laboratories reported full shut-downs of at least one month (Figure 1).
